# Assessing food-based strategies to address anaemia in pregnancy in rural plains Nepal: a mixed methods study

**DOI:** 10.1017/S0007114522003208

**Published:** 2023-07-28

**Authors:** Joanna Morrison, Romi Giri, Philip James, Abriti Arjyal, Chandani Kharel, Naomi Saville, Sushil Baral, Sara Hillman, Helen Harris-Fry

**Affiliations:** 1 UCL Institute for Global Health, 30 Guilford Street, London WC1N 1EH, UK; 2 Herd International, Thapathali, Kathmandu, Nepal; 3 London School of Hygiene and Tropical Medicine, Keppel St, London WC1E 7HT, UK; 4 UCL Institute for Women’s Health, 74 Huntley Street, London WC1E 6AU, UK

**Keywords:** Maternal, Nutrition, Community, Lay aetiology, Iron deficiency

## Abstract

Anaemia in pregnancy is a persistent health problem in Nepal and could be reduced through nutrition counselling and strengthened iron folic acid supplementation programmes. We analysed 24-hour diet recall data from 846 pregnant women in rural plains Nepal, using linear programming to identify the potential for optimised food-based strategies to increase iron adequacy. We then conducted qualitative research to analyse how anaemia was defined and recognised, how families used food-based strategies to address anaemia, and the acceptability of optimised food-based strategies. We did 16 interviews of recently pregnant mothers, three focus group discussions with fathers, three focus group discussions with mothers-in-law and four interviews with key informants. Dietary analyses showed optimised diets did not achieve 100 % of recommended iron intakes, but iron intakes could be doubled by increasing intakes of green leaves, egg and meat. Families sought to address anaemia through food-based strategies but were often unable to because of the perceived expense of providing an ‘energy-giving’ diet. Some foods were avoided because of religious or cultural taboos, or because they were low status and could evoke social consequences if eaten. There is a need for counselling to offer affordable ways for families to optimise iron adequacy. The participation of communities in tailoring advice to ensure cultural relevance and alignment with local norms is necessary to enable its effectiveness.

Anaemia in pregnancy affects 38 % of women and girls and is one of the main causes of maternal deaths and adverse pregnancy outcomes in low-and middle-income countries^(1,2)^. Intervening at the community level can help to reduce anaemia in pregnancy. Combining iron folic acid (IFA) supplementation with antenatal counselling and nutrition education programmes has been found to reduce the risk of maternal anaemia by 30 %^(3)^. For the counselling and nutrition education components to be effective, it is important that they are tailored to the context and culturally appropriate^(4)^. Qualitative research to understand local perceptions of anaemia and its causes, and perceptions of iron-rich foods, is important to design contextually appropriate interventions^(5)^.

In Nepal, anaemia in pregnancy remains a persistent problem. The latest Demographic and Health Survey (DHS) found that 46 % of pregnant women were anaemic despite relatively high coverage of IFA supplementation^(6)^. Side-effects and access to antenatal care can affect consumption of IFA, particularly for the most marginalised^(7,8)^. Consumption of a diverse diet is affected by factors such as cost, availability, preferences and gender-based discrimination which can mean that pregnant women are prevented from accessing micronutrient-rich foods^(8–13)^. Cultural beliefs about the amount of food that is good to eat during pregnancy may also affect anaemia. The practice of ‘eating down’ (i.e. purposefully eating less than normal) has been reported in South Asia, where pregnant women eat down because they believe that food will occupy the space for the baby to grow^(14)^, and others eat down because of aversion to food, lack of appetite or feeling unwell^(15,16)^. Cultural beliefs about ‘eating down’ to ensure an easier delivery of a small baby have also been documented in the plains of Nepal^(8)^.

Beliefs about which food is good to eat and which food is good to avoid in pregnancy are often related to the vedic conceptualisation of food as ‘hot’ or ‘cold’^(17–19)^. It is considered important to maintain equilibrium of hot and cold in the body and consumption of heating or cooling foods can alter this balance^(20,21)^. An imbalance is believed to cause illness^(11)^. Pregnancy is commonly regarded as a ‘hot’ state requiring avoidance of heating foods, such as wheat, millet, soybeans, milk, fish and chicken. Conversely postpartum is regarded as a cold state and requires avoidance of cold foods, such as corn roti, black gram, yoghurt and buffalo meat^(22)^. It has been suggested that the specific classification of foods as hot or cold depends on beliefs about the illnesses caused by consuming a particular food^(23,24)^. The hot-cold classification is also related to a persons’ constitution and temperature (of food and of the environment)^(25)^. It follows that if a person is in a cold environment, food which is cold (ice-cream, cold drinks) should be avoided. Food taboos during pregnancy are also related to religious and ethnic beliefs which can affect what is consumed. For example, avoidance of meat is common amongst Hindus^(26)^.

We report on findings from formative research in rural plains Nepal to design a culturally sensitive intervention to address anaemia in pregnancy. Our published findings about the factors affecting access to antenatal care, consumption and compliance to IFA, and access to and consumption of iron–rich foods demonstrate the complex and interacting personal, family and community factors which influence anaemia in pregnancy^(8)^. In this paper, we analyse the extent to which locally available diets can be optimised to improve iron intake using ‘Optifood’ a linear programming software, which identifies optimised diets within known constraints for specific population groups of interest^(27)^. We then analyse qualitative data on perceptions of anaemia and iron-rich food to analyse the extent to which dietary recommendations fit with cultural beliefs.

## Methods

### Setting

This study was conducted to inform the design of a nutrition counselling intervention aiming to reduce anaemia in pregnancy in plains (terai) of Nepal^(28)^. The terai makes up only 23 % of Nepal’s landmass, but around 50 % of the population live there. It shares an open border with Uttar Pradesh and Bihar in India and migration for work is common^(29)^. A history of border disputes and resettlement initiatives has led to residence of high proportions of Muslim, Hindu Indian origin and Hindu Tharu ethnic and religious groups. These groups are amongst the most marginalised in the country^(30,31)^ and have suffered from a history of political marginalisation. The area has a similarly low Human Development Index score to the mountains of Nepal, largely driven by poor education indicators. In 2016, 65 % of primary and 77 % of lower secondary school-aged children who were out-of-school were resident in the terai^(32)^.

The terai contains Nepal’s most fertile and productive land. The food supply and general dietary patterns are similar across the terai with the main diet constituting *daal bhat tarkari* (rice, spiced lentil soup and curry usually made with potatoes) and low intakes of fruits, vegetables and animal-source foods. Anaemia in pregnancy is most prevalent in the terai (52 %), compared to the mountains (35 %) or hills (29 %)^(33)^. This is particularly concerning as women living in the terai do not face the topographical barriers to access healthcare or food that are experienced in the mountains and hills, and household food security is higher in the terai than mountains and hills^(33)^. It is likely that the higher rates of anaemia in the terai are explained by a complex interplay of infection and restrictive gender norms. For instance, despite shorter travel times to health facilities in the terai, the DHS shows higher prevalence of childhood diarrhoea, poorer sanitation practices, lower access to deworming tablets in pregnancy and slightly lower compliance to IFA supplements in the terai^(33)^.

The terai has the highest levels of gender disparity in human development in Nepal^(34)^. Patrilocal marriage – whereby women move to their husband’s home after marriage – is common in Nepal. Patriarchal social norms are particularly restrictive in many parts of the terai. Women should show respect through subservience to their husband and husband’s family and they are under the protection and guardianship of that household^(13,35)^. Women’s mobility is strictly controlled after marriage and movement outside the household without permission is a common justification for intimate partner violence^(36)^. Restrictive gender norms limit women’s share of the household’s food^(8,13)^, and inadequate consumption of iron-rich foods and other micronutrients essential for healthy erythropoiesis and iron absorption have been reported amongst women in the terai^(37–39)^. This research was conducted in two terai provinces: Kapilvastu (Province 5), and Dhanusha and Mahottari (Province 2).

### Dietary sampling, data collection and analysis

#### Dietary sampling

The dietary analyses used a dietary dataset that had been collected between June and September 2015 in Province 2. The dietary data were collected in a random sample of 848 joint households enrolled in a pregnancy-focused four-arm trial in Dhanusha and Mahottari districts. The sample size was chosen to detect differences in intra-household allocation of dietary energy between each intervention and control^(40)^.

#### Dietary data collection and analysis

For the dietary analyses, we used a data set containing dietary intakes of 848 pregnant women. Diets were assessed using triplicate 24-hour dietary recalls between June and September 2015. This period covers pre-monsoon and monsoon season, so it includes a range of foods but may omit some foods available at other times of year. Interviewers conducted dietary recalls using a five-stage ‘multi-pass’ method, where participants are iteratively probed about their intakes over the previous 24 hours. Participants first provide a chronological list of all foods and drinks consumed the previous day, and then, in the next four ‘passes’, they describe time and place each food was consumed, review of forgotten foods list, recap the full list and finally provide portion size and food type specifics. Participants described portion sizes with the aid of a locally produced and validated photographic atlas. Data were entered electronically using a smartphone tool^(41)^.

We first used the dietary data to describe intakes of energy and protein, and intakes of micronutrients for which deficiency of both causes anaemia^(42)^ and is prevalent in Nepal^(9,43,44)^ i.e. iron, and vitamins A, C, B_2_ (riboflavin), B_6_, B_9_ (folate) and B_12_. To do this, we linked the food intakes and portion size data to a food composition table that had been previously compiled from multiple sources^(45–48)^ and calculated the nutritional intake for each pregnant women per day. Following Tooze *et al.* ^(49)^, we account for the wide within-person variance in dietary intakes by calculating ‘usual’ intakes of each nutrient. Usual intakes are the best linear unbiased predictor resulting from mixed-effects models using the triplicate recalls, where we treat people and Village Development Committees (administrative units) as random effects.

We then analysed the dietary data using the ‘Optifood’ linear programming software, which identifies optimised diets given known constraints for specific population groups of interest (pregnant women, in our case). For this, we generated three inputs for Optifood^(27,50,51)^. First, we generated a list of locally available foods by restricting the full list of 221 foods to those foods consumed by ≥ 10 % of the population and foods containing iron, giving a final list of fifty-four foods. This was done to ensure the optimised solution did not recommend intakes of very rarely consumed foods. Second, we calculated median daily portion size per food (g/d), and frequency of servings per week by 5th and 95th centiles, to define sensible upper and lower constraints on intakes for each food. Third, we used the food composition table and assumed 5 % bioavailability of iron given the high phytate diet in this context^(43)^. This information was entered into Optifood software, which then maximised iron given these constraints on intakes (i.e. median portion size and upper and lower bounds on frequency). We then adjusted the model parameters to inspect how the solution (i.e. recommendations and iron adequacy) change with varying constraints – particularly to compare vegetarian *v*. non-vegetarian diets and varying levels of animal-source foods that might be unaffordable and/or avoided by some subpopulations.

It is understood that the optimal ‘solution’ needs to be contextualised by review with local experts^(52)^, ideally by the population of interest themselves, to generate evidence-based, locally appropriate food-based recommendations. Optifood has been used extensively to generate food-based recommendations^(53)^, although, to our knowledge, mixed-methods approaches to integrate participants’ perspectives on their understanding and beliefs about diets and particular foods of interest, and dietary recommendations are rare^(54)^.

### Qualitative sampling, data collection and analysis

#### Qualitative sampling

For the qualitative research, we selected two rural and one urban municipality where we could sample community members from marginalised (Muslim and Dalit) groups. Within these municipalities, we purposively sampled mothers and mothers-in-law whose child/grandchild was aged ≤ 6 months to explore their recent experience of a pregnancy and community norms. These mothers and mothers-in-laws were from separate households. We sought to explore a breadth of experience, so we sampled primiparous and multiparous mothers, mothers from marginalised and less marginalised ethnic/caste groups (Table 1), and mothers from poor and better-off households. It was challenging to find many fathers with young children, so we sampled fathers with a child aged < 10 years to explore community norms and perceptions from a father’s perspective. We sought triangulation of findings about community norms through interviews with purposively sampled key informants and nurses from government health facilities. Key informants were identified after consulting with mothers-in-law, nurses and Female Community Health Volunteers and included non-governmental organisation employees, Female Community Health Volunteers and religious leaders.

**Table 1.** Ethnicity and religion of mothers, mothers-in-law and fathers

	Mothers (*n* 16)	Fathers (*n* 20)	Mothers-in-law (*n* 19)
Caste and ethnicity			
Low caste	6	8	11
Plains marginalised	5	7	7
Plains indigenous	4		
Plains	1	5	1
Religion			
Hindu	13	18	14
Muslim	3	2	5

#### Qualitative data collection and analysis

We recruited mothers, mothers-in-law and fathers through nurses and Female Community Health Volunteers. Researchers approached community members in their homes and nurses and key informants at their workplaces and took voluntary informed written or thumb-print consent to participate. No one refused to participate and data were collected locally in a place and time chosen by participants.

Between August and October 2019, we conducted sixteen semi-structured interviews (SSI) with mothers, four SSI with nurses, four key informant interviews, three focus group discussions (FGD) with twenty fathers in total and three FGD with nineteen mothers-in-law in total. Interviews and discussions were conducted using topic guides. Additionally, mothers and mothers-in-law discussed a list of iron-rich foods generated by the quantitative dietary analysis. This enabled us to discuss the consumption of those foods during the woman’s last pregnancy and the factors affecting consumption. Two trained, bilingual Awadhi Nepali-speaking female researchers who lived in Kapilvastu collected data. Topic guides and the food consumption list were developed in Nepali and Awadhi, and researchers were mentored by RG and JM who observed five interviews and one FGD.

Data were digitally recorded, transcribed into Nepali and translated to English for analysis. For quality assurance, random sections of 10 % of translations were checked against transcripts. Food consumption data about iron-rich food from mothers were tabulated and analysed by the religion and ethnicity of participants. We also compared their responses with data from mother-in-law FGD, although these data were not always collected consistently. For data about perceptions of anaemia and diet during pregnancy, we conducted descriptive content analysis, comparing data collected through different methods and from different respondent types^(55)^. RG and JM read transcripts and made notes independently before discussing and agreeing on preliminary codes. Codes were developed inductively and deductively by RG and JM and transcripts were coded in Nvivo v11 qualitative analysis software. Narrative descriptions of codes and findings from the food consumption list were discussed with the wider team.

## Results and discussion

### Diets to optimise iron intakes

Dietary intakes are described in Table 2. Intakes are low for many micronutrients, especially for vitamin A, riboflavin and vitamin B_12_ where mean intakes are below average requirements. However, we also see very wide standard deviations for all nutrients, reflecting strongly right-skewed distributions, most strikingly for iron and vitamin B_12_.

**Table 2.** Dietary intakes by pregnant women

	Estimated average requirement	Predicted usual intakes	
		Mean	(sd)
Energy (kJ)	–	9021	2092
Protein (g)	–	65	14
Iron (mg)	22·4	27	19
Vitamin A RAE (µg)	540	321	126
Vitamin C (mg)	80	100	46
Riboflavin (mg)	1·5	1·2	0·5
Folate (µg)	520	704	451
Vitamin B_12_ (µg)	2·2	0·5	0·5

Note: *n* 846 women. Intakes measured over 3 d; predicted usual intakes are the best linear unbiased predictors resulting from mixed-effects models using the repeated measures. Requirements are for pregnant women aged 19–30 year, assuming low iron absorption^(62)^.

Dietary patterns and the constraints used to optimise iron intakes are given in Table 3. iron-optimised diets are described in Table 4, with three scenarios of non-vegetarian diets and three scenarios of vegetarian diets. None of these optimised diets would result in pregnant women consuming 100 % of their recommended iron intake. This indicates that iron is a ‘problem nutrient’ in this context and that iron supplementation is needed, especially in pregnancy when deficiency poses the greatest health risks. Second, the iron levels of diets can be compared against the current average dietary patterns, which only met 32·2 % of the recommended nutrient intake for iron. This shows that some diets could substantially increase iron intake, especially diet patterns numbered 1 and 2 with more meat. On the other hand, iron content of the optimised vegetarian diets was still quite low. So, despite some sizable changes in the vegetarian diets recommended, these changes would not result in large improvements in iron adequacy.

**Table 3.** Constraints used within the linear programming modelling in Optifood software. Low servings = 5th centile; high servings = 95 % centile of dietary patterns from 848 households

Food group	Low servings/week	Median servings/week	High servings/week
Bakery and breakfast cereals	7	7	14
Beverages (non-dairy or blended dairy products)	2	7	9
Composites (mixed food groups)	2	2	14
Dairy products	7	7	21
Fruits	2	2	7
Grains and grain products	0·1	0·1	11
Legumes, nuts and seeds	3	3	10
Meat, fish and eggs	10	10	19
Miscellaneous	0·1	0·1	7
Savoury snacks	0·1	0·1	5
Starchy roots and other starchy plant foods	0·1	0·1	3
Vegetables	0	0·1	5

**Table 4.** Results from Optifood software aiming to optimise iron intake from available food sources, expressed as number of servings per week of a given food item

	Non-vegetarian dietary patterns						Vegetarian dietary patterns					
Diet pattern	1		2		3		4		5		6	
	Allows four meat portions, ten daal portions		Allows three meat portions, fourteen daal portions		Allows one meat portion		No meat or egg in diet		No meat, no egg but more green leafy vegetable portions allowed		No meat, but eggs allowed.	
Max % iron RNI	79·3		51·9		43·8		40·2		44·0		40·6	
Food type and frequency of intake per week												
	Food	*n*	Food	*n*	Food	*n*	Food	*n*	Food	*n*	Food	*n*
1	Spiced lentil soup (*daal*)	10	Spiced lentil soup (*daal*)	14	Spiced lentil soup (*daal*)	10	Spiced lentil soup (*daal*)	10	Spiced lentil soup (*daal*)	10	Spiced lentil soup (*daal*)	11
2	Deep fried vegetarian snack*	4	Wholewheat flatbread (*roti*)	9	Wholewheat flatbread (*roti*)	9	Wholewheat flatbread (*roti*)	9	Wholewheat flatbread (*roti*)	9	Wholewheat flatbread (*roti*)	9
3	Egg (chicken)	4	Deep fried vegetarian snack*	5	Green leafy vegetables†	5	Green leafy vegetables†	5	Green leafy vegetables†	10	Egg (chicken)	7
4	Green leafy vegetables†	5	Green leafy vegetables†	5	Flaxseed	4	Deep fried vegetarian snack*	5	Deep fried vegetarian snack*	5	Green leafy vegetables†	5
5	Pigeon curry	4	Aubergine curry	4	Milk	2	Fried potato (*Bhujiya*)	4	Fried potato (*Bhujiya*)	4	Deep fried vegetarian snack*	5
6	Aubergine curry	5	Pigeon curry	3	Pigeon curry	1	*Arikoch* curry‡	4	Aubergine curry	4	Aubergine curry	4

RNI = recommended nutrient intake.

* For example *pakora*, *aluchap*, *taruwa*, *kachuri.*

† Cooked with oil, salt and chilli (*sag*).

‡ Green leaves fried in ball shape.

Taking these different optimised scenarios together, we can see that increased consumption of meat, egg and green-leafy vegetables is recommended, which is relatively unsurprising given their iron content. Aubergine curry is recommended not for the iron content but because aubergine curries often contain green leafy vegetables. This can be interpreted as a recommendation to consume mixed vegetable curry with green leaves.

The optimised diets replace the main staple food (rice) with flatbread made from whole wheat flour. Rice is a major constituent of the diet, is culturally important in this context and is produced locally, so a recommendation to make this dietary change is unlikely to be successful. Further modelling could compare the relative gains of this dietary shift, although, given the small increases in iron adequacy of the optimised vegetarian diets compared with current dietary patterns, we can see that recommendation to swap the main staple would have both minimal impact and low feasibility.

Some fried foods were recommended (bhujia, pakora). Although the primary objective is to maximise iron intakes in our case, the outputs need to be interpreted holistically, to consider the rising prevalence of overweight in Nepal. Dietary recommendations could therefore suggest healthier recipes for these foods, that contain less salt and oil.

It is clear from our description of the linear programming results that dietary recommendations cannot be developed without knowledge of the context for which the recommendations are intended. In our case, the effectiveness of food-based interventions to reduce anaemia will depend on families recognising anaemia and being willing and able to modify their diets to increase their iron content. Appropriately designed interventions need to be designed with an understanding of the perceptions of anaemia symptoms, causes and effects and perceptions of iron-rich foods. We explore this in the next section.

### Perceptions of anaemia

#### Defining anaemia

Participants in qualitative interviews and group discussions described anaemia as a lack of blood in the body which causes weakness, dizziness and tingling in hands and feet. They described women with anaemia as ‘weak’, ‘thin,’ ‘lean’ and ‘unable to walk’: ‘It is difficult for (a woman with anaemia) to walk. Her face looks dejected and becomes yellow…You get dizzy…It is difficult to even stand up after sitting’ (Mothers-in-law 102 FGD). Women with anaemia were described having blurred vision, sunken eyes and a yellow or black appearance: ‘They look thin and are dark-skinned. Their face turns black…They look lazy and yellowish’ (Fathers 105 FGD). Conversely, those who were healthy looked ‘green’: ‘(Women with anaemia) look weak. Only if you get balanced food, does your body look green [healthy]’ (Mother 101 SSI). One mother (104 SSI) described a woman with anaemia as looking like a lizard. Swelling of the body, particularly of hands, legs and feet, was commonly reported. Mothers who had anaemia during their pregnancy also reported having stomach pains, pain in their joints, shivers and loss of appetite. Qualitative research from Bolivia, Burkina Faso, Guatemala, Honduras, India, Indonesia, Malawi and Pakistan^(56)^ found similarities to our data in the way that women described anaemia, referring to it through the description of symptoms instead of as a specific illness.

#### Causes of anaemia

One community key informant and a group of husbands believed that women need more blood during pregnancy – because of the loss of blood that occurred during delivery, and because they are making a baby: ‘The pregnant women have lack of blood in their body because there is a development of two bodies inside one body’ (Fathers 106 FGD). Participants said that food was required to make blood, and therefore the lack of blood in the body was caused by inadequate consumption of water and food. A group of mothers-in-law (102) said that without adequate food: ‘There will be swelling in the body and there will be no formation of blood in the body’ (Mothers-in-law 102 FGD). Inadequacy of food or water was believed to cause bodily imbalance, which would lead to less blood and weakness: ‘There will be inadequacy of blood if the food and water are not balanced. There will be weakness in the body’ (Mother 108 SSI). Not eating on time, and not eating ‘properly’ were also commonly noted as causes of anaemia, and this was linked to the economic status of the family: ‘There will be low blood if the food pattern is not proper because of their economic condition’ (Mothers-in-law 101 FGD). Part of eating ‘properly’ was eating foods that were perceived to give energy, and foods that were perceived to be rich in vitamins. A lack of vitamins was thought to cause anaemia. Other contributing factors were mentioned including stress during pregnancy (a mother), short birth intervals (fathers), and resting too much (a mother and both groups of fathers). There was diversity in lay aetiologies of anaemia, with some conflation of cause with symptoms.

#### Anaemia prevention

Mothers, mothers-in-law and fathers felt that prevention of anaemia was primarily linked to adequate and regular diet and water consumption. Despite this, most mothers said that their diet did not change during pregnancy, and they ate combinations of daal, rice, curried vegetables and roti as finances allowed: ‘If you don’t eat enough food, then you won’t have enough blood. The food pattern we eat is based on the income we have. It’s not enough just to know what we should eat. Where will we get it from?’ (Mother SSI 105). Mothers found it difficult to alter their eating habits to accommodate their pregnancy because of family commitments and a general lack of food: ‘If you don’t eat at the right time then there will be weakness. We need to eat food on time. I find it difficult to eat food in front of my children. I have four children. How can I eat all by myself and not give them?’ (Mother SSI 101). ‘There were no changes in my food pattern because we don’t have money. It was just daal, rice and vegetable curry. If I wanted to buy something in the market my husband told me that we didn’t have money. What changes (in my diet) could there be? I just ate the same dry food’ (Mother SSI 102). The inability to alter diets to prevent anaemia because of poverty has also been reported in recent research on anaemia in pregnancy in India^(57,58)^.

Eating meat and fish to prevent anaemia was discussed by fathers, community leaders and only a few mothers. Fathers also said that consuming eggs, beans and chickpeas could help prevent anaemia. Fruit, curd, milk and vegetables were commonly discussed by mothers and mothers-in-law. Recommended food to prevent anaemia was often red in colour, probably because of the analogy with blood. Apples, pomegranate and beetroot were specifically mentioned, and these foods tended to be expensive: ‘(Health workers) told me to eat pomegranates and apples. I ate it so that I won’t have weakness’ (Mother SSI 104). Young *et al.* also found a cultural association between red food as preventative of anaemia in Zanzibar^(59)^. Interventions could encourage the substitution of some foods that are expensive and not iron-rich (although perceived to be), such as apples and pomegranate, for foods that are richer in iron.

These data demonstrate a clear understanding of the nutritional value of dietary diversity. Dietary recommendations developed from the Optifood analyses – to increase intakes of meat, egg and green leafy vegetables – generally align with local beliefs of food-based strategies to prevent anaemia. On the other hand, some foods perceived to be iron-rich are iron inhibitors (curd, milk) but otherwise nutritionally important, so dietary recommendations could encourage eating these foods at different times of day from the iron-rich foods.

### Perceptions of Iron-rich food

#### Iron-rich food consumption during pregnancy

The consumption of different types of animal foods was shaped by religious beliefs as well as caste and wealth. Despite our modelling of both vegetarian and non-vegetarian diets, chicken was eaten by fifteen out of sixteen mothers during pregnancy, although one of these mothers said that it made her nauseous. Fifteen mothers ate fish, although one said that she only ate it once or twice because it was expensive. Buffalo and pork or wild boar were not eaten by many mothers. Fourteen out of sixteen mothers did not eat pork: ‘We don’t eat this in our family. It is a dirty animal’ (Mother SSI 103). Many said that it was forbidden by God/Allah, and some stated that only the lowest castes consumed this type of meat. One mother said: ‘We are not allowed to eat (pork) in our Hindu culture. Dalit (low caste) people eat that. They eat in Chamar and Pasha (castes)’ (Mother SSI 116). Only one mother had eaten buffalo, and others said it was forbidden by their culture or religion. A few stated that Muslims could eat buffalo but none of the Muslim mothers in our study ate it. One Muslim mother said: ‘No one in our family eats buff. It is not allowed in this community’ (Mother SSI 110). Ten mothers had eaten mutton (goat meat) during their pregnancy and those who had not eaten it did not like it or found it difficult to digest. It was thought to smell bad amongst a few mothers, and two mothers who did not like it also said it was expensive ‘I don’t like it. You need money for this too.’ (Mother SSI 104). There were no stated taboos around eating mutton. Pigeon was also not taboo, but not widely available and only seven mothers had eaten pigeon: ‘No, I didn’t (eat it). We don’t keep pigeons in our village. No one brings it in our home too, so we don’t eat it’. (Mother SSI 102). One woman said that it was too expensive for her family to eat.

This indicates that although the dietary recommendations from the optimised analyses included pigeon meat, such a specific suggestion may be less useful and popular. Substituting a recommendation for ‘pigeon meat’ specifically for ‘any meat of their preference’ would give similar iron levels and could be more popular amongst families. For example, goat, duck and pigeon meat all have similar iron content.

Snails had not been consumed by any mother during her pregnancy, despite being an iron-rich food. There was a taboo about eating snails. Three mothers explicitly said that it was ‘not allowed’ to eat snails and one said: ‘We do not eat that. No one in this community eats that. It is humiliating if anyone sees us eating snails and everyone talks behind our backs about it’ (Mother SSI 110). One group of mothers-in-law also mentioned that snails were not eaten because people would make fun of you. Four mothers found the idea of eating snails to be disgusting, and two mothers and a group of mothers-in-law said that only people who were ill, specifically people with tuberculosis ate snails: ‘Only sick patients eat it. I didn’t have any problem. So, I didn’t eat it’ (Mothers-in-law FGD 115). One mother and a group of mothers-in-law discussed the fact that eating snails was place specific: ‘We don’t eat snails in my maternal home. I heard about it when I came here (after marriage) but I don’t know snails’ (Mother SSI 101). This mother said that those belonging to the Tharu (Hindu indigenous) ethnic group eat snails. Mothers-in-law said: ‘I ate so many when I was young…but after I came here I haven’t eaten them…We get energy if we eat this but we don’t eat it’ (Mothers-in-law FGD 101).

Food taboos for buffalo, pork and snails were based on religious and cultural beliefs, with Muslims in Hindu areas conforming to dominant Hindu social norms by not eating buffalo, and Tharu’s conforming to dominant cultural norms by not eating snails. These taboos were followed to protect women and their babies^(60)^ and corresponded with women’s personal preferences. Therefore, they would not be amenable to change. Food taboos were also associated with the social acceptability of the food, and the status of people who were thought to eat that food. High caste Hindu’s often follow a vegetarian diet, and the sanscritisation of eating practices among lower castes or other disadvantaged ethnic groups has been documented as a route to social mobility^(61)^. The status of foods is an important determinant of consumption.

All mothers interviewed had eaten green leafy vegetables, daal and other pulses except one who did not like them whilst pregnant. All fourteen mothers who were asked about eggs had eaten them in their last pregnancy. Only five mothers had eaten sprouted beans and pulses during pregnancy, and most were unaware that they were iron-rich or good to eat during pregnancy.

Iron-inhibiting dairy foods were commonly consumed. Most mothers had drunk milk and eaten yoghurt during their pregnancy. Of the mothers who had not drunk milk, one woman said that her family did not bring it for her and the other said that her buffalo did not give milk while she was pregnant. Ten mothers had drunk buttermilk. For some it was not available at home, and two mothers did not like it. Paneer was only consumed by five mothers as it was expensive and rarely brought to their home. *Ghee* (clarified butter) was also only consumed by five mothers, as others did not like it, found it too expensive or it was not available at home. Mothers-in-law said that ghee helped the delivery, enabling the baby to be born at home, which was preferred. Other milk products such as *Khuwa* (milk evaporated into solid form), rice pudding and sweets were eaten by some mothers. Mothers-in-law said: ‘You get energy after you eat milk, yoghurt and ghee and your body smells pleasant, but your body smells bad if you eat meat and fish’ (FGD 101). Dairy foods were commonly consumed (as a drink, sweets, puddings), and therefore a recommendation to consume these items at different times from iron-rich foods might be feasible.

There was some discussion of what should be eaten during pregnancy in FGD – unrelated to anaemia – and there was variation within and between FGDs. Some common ideas were that pregnant women prefer foods that are spicy and sour, and that they should not eat stale food. Another commonality was that food should not be too much of one taste – for example, ‘not too spicy’ and ‘not too sour’ so that the pregnant woman could digest it. This group discussion among a group of mothers-in-law exemplifies this: ‘You shouldn’t eat noodles. You also should not eat egg because the hot temperature is harmful…You won’t have any problem if you eat one egg. …Yes, it won’t be a problem if you only eat one egg but mostly, (pregnant women) want to eat at both mealtimes which is harmful…How can we feed them at both mealtimes? No one asks for eggs twice…Those who like it and can digest will eat…You should not eat nor too hot nor too cold food. You should eat food of medium temperature that your stomach can digest’ (Mothers-in-law FGD 102).

These food taboos appeared to be associated with maintaining bodily balance, and therefore depended somewhat on how much the pregnant woman could tolerate, or digest, and did not appear to overly restrict their access to iron-rich food. Other studies have found that pregnant women are often advised to avoid ‘heating’ foods, such as milk^(58)^, but we did not find clear patterns in this regard. Other research has found that knowledge of food taboos does not directly relate to practise, and practise depends on the perceived sanctions for eating these foods^(60)^. It is important that women and families have access to information about how to increase iron in their diets from a variety of foods and food-based strategies, so that they can choose strategies to address anaemia that they feel comfortable with.

### Limitations

Diet optimisation analyses were conducted using data from a different district to Kapilvastu and select times of year (June to September). So, we may have missed Kapilvastu-specific food items and recipes and we are unable to examine the seasonality of dietary iron supply. Qualitative results indicate that cost was a major barrier for many to purchase iron-rich diets, but we were unable to run cost minimisation analyses due to a lack of price data. Further work could consider the cost of these dietary recommendations and identify foods with the least cost per mg iron, to help tailor advice for poorer families. FGD participants were from different ethnic and religious groups and therefore we were unable to explore cultural differences in depth, but we were able to analyse data from SSIs with mothers by ethnicity and religious group which helped us understand where variation may occur. We analysed data that had been translated from Awadhi to Nepali and then into English; meaning may have been lost in this process. Translators were trained to maintain Awadhi words where they did not lend themselves easily to translation, and the analysis team conferred with Awadhi-speaking researchers for clarification.

### Conclusion

Addressing anaemia in pregnancy is a global priority, and strategies which reach the poorest and most marginalised groups are necessary to improve maternal and newborn health. We have presented an analysis of iron-optimised diets in rural plains Nepal and considered what this means in the local context. Iron is a problem nutrient: 100 % adequacy cannot be achieved with sensible intake levels of locally available foods. However, iron adequacy could be substantially improved (approximately doubled) by incorporating more meat, egg and green leafy vegetables into the diet. We found that there was awareness of the problem of anaemia and its adverse health consequences for mothers and babies. Families sought to address anaemia through food-based strategies but were often unable to do so because of the perceived expense of providing an ‘energy-giving’ diet. Avoidance of some iron-rich foods was based on religious and cultural taboos, and some foods were avoided as they were low status and could evoke social consequences if eaten. However, there was a general understanding of the nutritional importance of diverse diets, and many could identify iron-rich foods that aligned with the foods recommended in the iron-optimisation analyses. Our research makes an important contribution to the literature as we demonstrate how to integrate qualitative research findings about local context with recommendations from Optifood software, to tailor advice about increasing iron intake. Tailoring can increase the relevance and acceptability of food-based recommendations to address anaemia in pregnancy.

Utilising community-based culturally appropriate nutrition counselling combined with strengthened IFA programming has the potential to reduce anaemia in pregnancy. We have shown that dietary patterns could be modified to increase iron adequacy, and that communities in rural plains of Nepal already recognise the signs of anaemia and seek food-based strategies to prevention, within the constraints of poverty. There is a need for counselling to offer affordable ways for families to increase iron absorption and emphasise early intervention when a woman is showing signs of anaemia. The participation of communities in tailoring dietary advice is necessary to ensure alignment with local norms and thereby increase the likelihood of its effectiveness.
